# Microbial Communities Affected by Hydraulic Fracturing and Environmental Factors within an In Situ Coal Reservoir

**DOI:** 10.3390/microorganisms11071657

**Published:** 2023-06-25

**Authors:** Yang Li, Jian Chen, Shuheng Tang, Zhaodong Xi

**Affiliations:** 1 School of Earth and Environment, Anhui University of Science and Technology, Huainan 232001, China; 2 The Key Laboratory of Universities in Anhui Province for Prevention of Mine Geological Disasters, Anhui University of Science and Technology, Huainan 232001, China; 3 Key Laboratory of Petroleum Resources and Prospecting, China University of Petroleum, Beijing 102249, China; 4 School of Energy Resource, China University of Geosciences, Beijing 100083, China; 5 Key Laboratory of Marine Reservoir Evolution and Hydrocarbon Enrichment Mechanism, Ministry of Education, Beijing 100083, China; 6 Key Laboratory of Strategy Evaluation for Shale Gas, Ministry of Land and Resources, Beijing 100083, China

**Keywords:** coalbed methane, biogeochemistry, methanogens, microbial diversity, the Qinshui Basin

## Abstract

The rise of coalbed methane bioengineering enables the conversion and utilization of carbon dioxide through microbial action and the carbon cycle. The environment of underground coal reservoirs is the result of a comprehensive effort by microorganisms. Some studies on reservoir microorganisms have progressed in laboratory conditions. However, it does not replicate the interaction between microorganisms and the environment on site. Hydraulic fracturing is an engineering technology to improve the natural permeability of tight reservoirs and is also a prerequisite for increasing biomethane production. In addition to expanding the pore and fracture systems of coal reservoirs, hydraulic fracturing also improves the living conditions of microbial communities in underground space. The characteristics of microbial communities in the reservoir after hydraulic fracturing are unclear. To this end, we applied the 16S rRNA sequencing technique to coalbed methane production water after hydraulic fracturing south of the Qinshui Basin to analyze the microbial response of the hydraulic fracturing process in the coal reservoir. The diversity of microbial communities associated with organic degradation was improved after hydraulic fracturing in the coal reservoir. The proportion of Actinobacteria in the reservoir water of the study area increased significantly, and the abundance of Aminicenantes and Planctomycetes increased, which do not exist in non-fracturing coalbed methane wells or exist at very low abundance. There are different types of methanogens in the study area, especially in fracturing wells. Ecological factors also determine the metabolic pathway of methanogens in coal seams. After hydraulic fracturing, the impact on the reservoir’s microbial communities remains within months. Hydraulic fracturing can strengthen the carbon circulation process, thereby enhancing the block’s methane and carbon dioxide circulation. The study provides a unique theoretical basis for microbially enhanced coalbed methane.

## 1. Introduction

Coalbed methane (CBM), which is released from coal seams, is a significant energy reserve for unconventional natural gas worldwide. The rapid expansion of CBM exploitation and utilization has effectively reduced greenhouse gas emissions, improved the energy supply structure, and met low-carbon environmental requirements. It has been confirmed that the origin of CBM is either thermogenic or biological [1,2,3]. Laboratory culture research and in situ studies have shown that continuous and active microbial communities generally exist in coal reservoirs. Active methanogenesis resulting from microbial activities, especially at shallow depths, still occurs mainly in some sedimentary basins, such as the Powder River Basin in North America and the Surat Basin in Australia. Most early observations of CBM reservoirs agree that biogenic CBM in shallow coal seams is produced from methanogenic microbial communities triggered by surface runoff and meteoric water that are identified by their stable methane isotope characteristics [4,5,6]. In addition to bringing microbial communities into coal reservoirs, water sources have been identified as carrying nutrients and electron acceptors that stimulate methanogenesis in CBM reservoirs. The study of geological microbiology has developed a unified understanding. In theory, increased methane production in coal seams can be achieved by stimulating microbial metabolism [7].

Uses of accelerating microbial action and increasing CBM resources have attracted increasing attention. In many cases, microbially enhanced CBM exploration can be used as a more environmentally friendly and economical energy source compared to coal as well as to extend CBM’s productive lifespans [8,9]. Shimizu et al. (2007) reported microbial communities related to coalbed methane in northern Japan [10]. Subsequently, microbial communities in coalbed methane reservoirs in the United States, Australia, Canada, and China were studied. In most studies, converting complex coal with recalcitrant macromolecules into biomethane requires some methods in laboratory conditions, such as temperature, pH, trace elements, coal particle size, and macerals were optimized, and some stimulation, such as adding beneficial nutrients to activate the metabolic activity of microbial communities [11]. In addition, the domestication of microorganisms and the introduction of exogenous microorganisms into laboratory environments were identified as feasible. These methods improve the environmental adaptability of these microorganisms and accelerate the process of degrading coal to methane [12]. However, coal biodegradation requires the cooperation of microbial communities with different metabolic characteristics, such as hydrolysis bacteria and other types of microbes [9].

Although some progress has been made in laboratory research to accelerate the biogenic methane production process, the level of increased CBM production in the field is not satisfactory. In addition, methanogenesis has been confirmed to likely occur inside permeable fractures and macropores rather than mesopores and micropores in the coal matrix [12]. As an effective technique to improve the natural permeability of tight reservoirs, hydraulic fracturing is considered to be a precondition for implementing microbial stimulation measures. In addition, to enhance the fracture and pore network of the coal seam for beneficial material delivery, the generated fractures derived from hydraulic fracturing will allow more significant microbial colonization and physical access to coal surface areas [6]. Most studies have focused on microbial communities during the microbially enhanced CBM process through culture-dependent techniques. Despite this research being particularly successful in laboratory conditions, our understanding of microbial communities’ exposure to in situ environments and hydrofracturing is limited, which hinders our strategies to increase native microorganisms’ access to coal and produce additional methane. Microbial communities have unique gene expression and physiological functions in anaerobic coal reservoirs relative to the species under laboratory conditions [4,6,8].

According to the metabolic behavior of underground microbial communities, if the coal reservoir is modified, it may stimulate the microorganism to continuously produce biomethane to prolong the production life of depleted coalbed methane wells. Hydraulic fracturing is an effective method to improve oil and gas production in tight reservoirs. The fracture system provides sufficient space for the survival and propagation of microorganisms and is also conducive to increasing the permeability of coal seams and material transport. Therefore, understanding the compositional response of microbial communities in the reservoir after hydraulic fracturing is the key to improving in situ biomethane production [13]. Hydraulic fracturing, chemical compositions, and certain additives such as gelling agents, proppants/breakers, and biocides of hydraulic fracturing fluids certainly affect the metabolism of microorganisms during the hydraulic fracturing process [13,14,15]. However, how hydraulic fracturing alters the metabolic reconstruction of microbial populations in CBM reservoirs is unknown. Meanwhile, for the discharge of fracturing fluids, a breaker is essential to depolymerize the gelling agents [14,15].

The primary objective of this study was to analyze dynamic microbial responses in situ during hydrofracture stimulation. The process of 16S rRNA sequencing can be used to estimate microbial community composition and metabolisms and the relationship between microbial communities and their living environments. Therefore, to study the dynamic characteristics of microorganisms in situ, CBM co-produced water was subjected to 16S rRNA sequencing to identify microbial compositions and metabolic functions in CBM reservoir environments. Samples were systematically collected through hydraulic fracturing. The functional potential of microbial communities was investigated based on the metagenome data set. We could reconstruct some unknown and extensive biological cycles by combining geochemical data. In addition, the study provides a unique theoretical basis for microbially enhanced CBM.

## 2. Materials and Methods

### 2.1. Study Area

**Figure 1:**
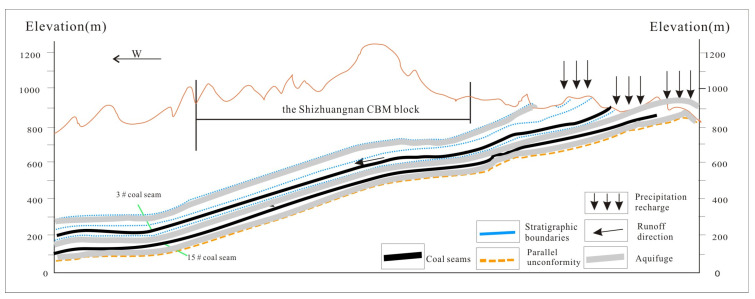
Hydrogeological conditions and regional structure in the Shizhuangnan block of the Qinshui Basin.

**Figure 2:**
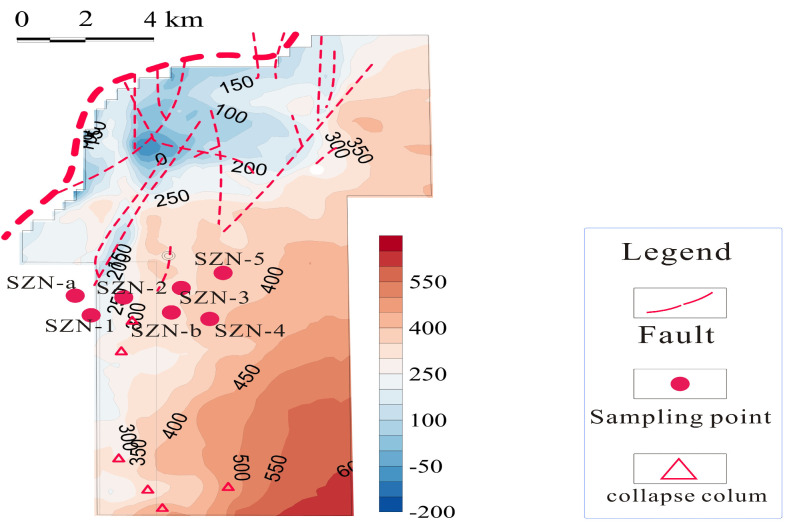
Schematic outline of the Shizhuangnan Block and sampling points.

The commercial development of coalbed methane in the southern Qinshui Basin is a demonstration area for the exploitation and utilization of coalbed methane resources in China [16,17,18]. The Shizhuangnan Block south of the Qinshui Basin has Carboniferous-Permian coal-bearing strata, including Taiyuan and Shanxi Formation in the Upper Pennsylvanian and Lower Permian [5,12]. As shown in Figure 1 and Figure 2, the Shizhuangnan Block is a monoclinic structure with almost north–south distribution and west-dipping. Hydrogeological conditions are controlled by the regional system, making it a relatively closed hydrogeological unit and a natural laboratory for studying microbial action. (Please refer to the relevant documents for the detailed geological and hydrological background of the study area [5,12,17].) SitouFault and other normal faults developed northwest of the Shizhuangnan Block have a sealing effect on the reservoir. The elevation of the No. 3 coal seam gradually decreases from east to west [5,6]. Total dissolved solids (TDS) in the reservoir west of the study area are generally high, and it is also the main distribution area for high-yield wells [12,17]. The construction parameters of gas fracturing in the Shizhuangnan Block are similar, and the active hydraulic fracturing fluid is used. Because the injected fluid is clean water with very low viscosity, damage and pollution to the coalbed methane reservoir is reduced. This study collected water samples from coalbed methane wells in the Shizhuangnan Block of the Qinshui Basin for geochemical testing and 16S rRNA sequencing, focusing on the evolution of abundance, diversity, and metabolic function in the process of microbial hydraulic fracturing of reservoirs in the Shizhuangnan Block.

### 2.2. Collection of Samples and Geochemical Analysis

As shown in Figure 2, water samples were collected from 7 previously sampled coalbed methane production wells in the Shizhuangnan Block for 16S rRNA sequencing and geochemical testing. Temperature and pH were measured on site. Water samples for geochemical testing and 16S rRNA sequencing were collected within one week. The unfractured CBM wells were named SZN-1, SZN-2, SZN-3, SZN-4, and SZN-5. Two samples were taken from wells after fracturing for 3 months and 5 months, which were named SNZ-a and SNZ-b, respectively. In addition, the selected well sites had good gas production. Pipes collected water samples discharged from the reservoir water at a high flow rate to avoid interference with residual water in the pipes. Before sampling, temperature, pH, and conductivity were measured on-site with a portable Accume multimeter to ensure data accuracy. Water from the coalbed methane well was filtered through the filter. Most solid particles were filtered to avoid interfering with subsequent tests. Water samples were collected with a 2 L sampling bottle and a 50 mL centrifuge tube for geochemical testing and 16S rRNA sequencing. The sampling bottle was filled with a water sample and sealed to ensure that the air in the bottle was discharged. The container was rinsed with the collected water sample three times before sampling. To reduce uncertainty or human error, 5 samples were collected at each sampling point for 16S rRNA sequencing, and 5 samples from each sampling site were mixed to meet the required biomass. Prior to DNA extraction, samples used for 16S rRNA were quickly frozen with dry ice. The ion test was based on the coal industry standard “Water Quality Analysis of Coal Mine Water” (MT/T894-2000). Cations and anions were tested via inductively coupled plasma atomic emission spectroscopy and ion chromatography. TDS is the sum of major anions and cations.

### 2.3. DNA Extraction and Sequencing Analysis

To ensure the integrity of microbial communities, the microbial samples collected from the study area were stored in a 4 °C incubator with dry ice until the completion of DNA extraction. The DNA for each sample was isolated with the PowerWater DNA Isolation Kit (MO BIO Laboratories, Carlsbad, CA, USA) according to the manufacturer’s specifications. DNA was measured with a Qubit Fluorometer using the Qubit dsDNA BR Assay kit (Invitrogen, Waltham, MA, USA), where a 1% agarose gel aliquot was used to ensure quality.

The amplified sub-library was amplified according to the 16S Sequencing Library Preparation Protocol. The V3–V4 region of the 16S rRNA was amplified with degenerate PCR primers 341F (5′-ACTCCTACGGGAGGCAGCAG-3′) and 806R (5′-GGACTACHVGGGTWTCTAAT-3′). Volumes of 30 ng of template, fusion PCR primer, and PCR master mix were added in a 50 L reactor, and PCR enrichment was performed.

The PCR amplification was measured under the reaction cycle as follows: 94 °C for 3 min, 30 cycles of 94 °C for 30 s, 56 °C for 45 s, 72 °C for 45 s, and 72 °C for 10 min of extension. To purify and elute to obtain PCR products, Am-pureXP beads and elution buffer were used.

The paired-end Illumina reads’ quality was examined using FastQC. To remove ambiguous and low-quality bases, raw reads were filtered. Then, paired-end reads were added via FLASH to obtain the tags. These tags were grouped into OTUs (Operational Taxonomic Units) with a 97% cut-off value by using the UPARSE program. Using the RDP classifier, OTU representative sequences were classified. OTUs were created to classify the sequence according to the needs of phylogeny or population genetics to analyze a taxonomic unit to understand the number of strains and genera in the sequencing results. To obtain species classification information for each specific OTU, it was necessary to classify and analyze the OTU representative sequences and count the microbial composition of the sample at different levels.

PCA (Principal Component Analysis) was used to simplify multivariate data, and hierarchical cluster analysis was used to analyze microbial communities in the sample. RDA (Redundancy Analysis) is a linear model method for studying microbial conditions, and it was used to analyze the impact of many environmental factors on microbial structure. RDA and PCA were analyzed with R software.

## 3. Results and Discussion

### 3.1. Geochemistry and Microbial Structure

Seven CBM sampling wells were located in the Shizhuangnan Block south of the Qinshui Basin. The data used in this study are shown in Table 1. Geochemical parameters such as SO_4_^2−^ and NO_3_^−^ may be the basis for the microbial action and biogeochemical cycle of the reservoir in the study area. The microbial communities of the reservoir in the Qinshui Basin were investigated. However, the dynamic change and response of microbial communities in situ after hydraulic fracturing had not been thoroughly studied. To this end, samples were taken from wells SZN-a and SZN-b 3 and 5 months after fracturing and from five nearby control wells (SZN-1, SZN-2, SZN-3, SZN-4, SZN-5), and 16S rRNA sequencing was used to analyze the impact of hydraulic fracturing on microbial community abundance, species, and metabolic function. Previous research has shown that SZN-a and SZN-b pre-fracking wells are very similar to control wells in terms of geographic distribution, coalbed methane production capacity, and reservoir biogeochemistry [5,12,17]. SZN-a and SZN-b were sampled 3 and 5 months after fracturing, respectively, so they could be used to determine the impact of different fracturing periods on microbial communities in the underground reservoir.

**Figure 3:**
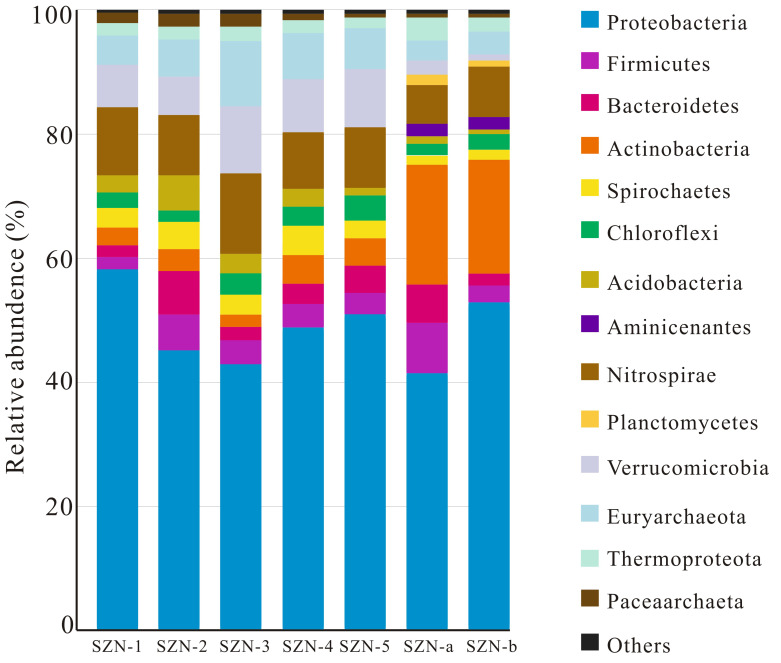
Microbes’ relative abundance at the phylum level of the water samples collected from coal reservoirs in the Shizhuangnan Block. (Phylum abundances of at least 1% were included; all remaining populations are indicated as “others”).

**Figure 4:**
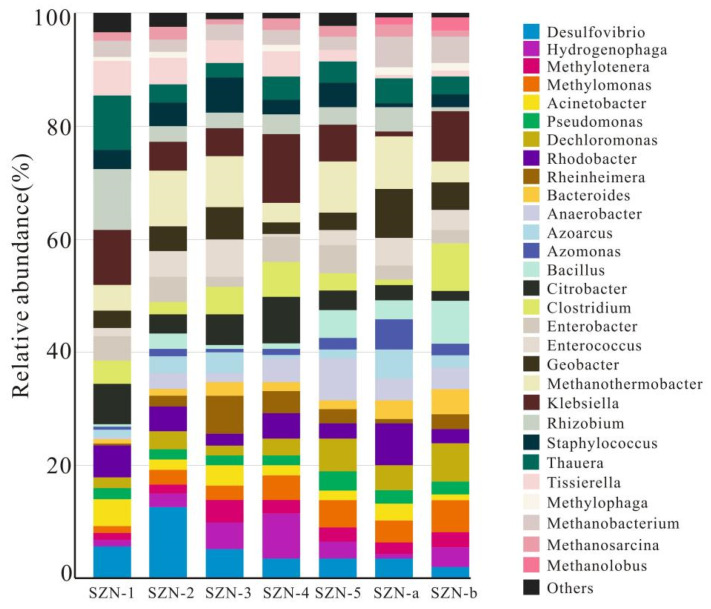
Microbes’ relative abundance at the genus level of the water samples collected from coal reservoirs in the Shizhuangnan Block. (Phylum abundances of at least 1% were included; all remaining populations are indicated as “others”).

As shown in Figure 3, sequencing results showed microbial communities in each water sample at the phylum level. In the control group (SZN-1, SZN-2, SZN-3, SZN-4, and SZN-5), microbial communities mainly included *Acidobacteria*, *Actinobacteria*, *Bacteroidetes*, *Chloroflexi*, *Firmicutes*, *Nitrospirae*, *Proteobacteria*, *Spirochaetes*, and *Verrucomicrobia*. In SZN-1, SZN-2, SZN-3, SZN-4, and SZN-5, the dominant bacteria were *Proteobacteria*, *Firmicutes*, *Bacteroidetes*, *Actinobacteria*, *Spirochaetes*, and *Chloroflexi*. In particular, *Proteobacteria* accounted for the largest proportion of all water samples. The microbial compositions of the fracturing wells (SZN-a, SZN-b) were significantly different from that of the control group. The ratio of *Actinobacteria* in reservoir water was dramatically increased at the phylum level, and new enrichment of *Aminicenantes* and *Planctomycetes* appeared. As shown in Figure 4, the microbial composition of the control group was similar at the phylum level. Nevertheless, microbial communities in each water sample had some species and abundance differences at the genus level.

As shown in Figure 3, at the phylum level, *Euryarchaeota*, *Nitrosopumilus*, and *Paceaarchaeta* were present in all water samples, but *Euryarchaeota* was the dominant archaea type. Other types of archaea were far from *Euryarchaeota*. Methanogens can only use simple organic substances with no more than two carbon atoms [19,20,21]. According to the different substrates available, methanogens can be divided into hydrogenotrophic, acetoclastic, and methylotrophic methanogens [22,23]. As shown in Figure 4, *Methanobacterium* and *Methanosarcina* were present in the control group at the genus level. In fracturing wells SZN-a and SZN-b, the proportion of hydrogenotrophic methanogens *Methanobacterium* increased, and a new methanogen (methylotrophic *Methanolobus*) appeared.

**Figure 5:**
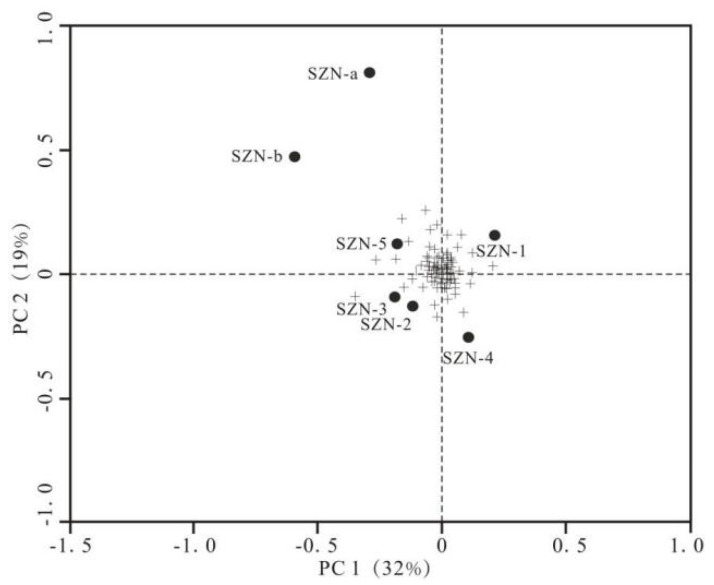
PCA of OTU for each water sample. (Plus signs are individual OTUs, and dots are water samples).

To analyze differences in microbial composition, PCA was used to analyze the apparent microbial communities’ separation between samples. As shown in Figure 5, the similarity of microbial communities in water samples from the control group (SZN-1, SZN-2, SZN-3, SZN-4, and SZN-5) was high. Microbial species in fracturing wells (SZN-a and SZN-b) were higher than in the control group. The difference between the fracturing well and the control wells was caused by differences in individual microbial abundance, such as the increase in Actinobacteria abundance in the fracturing well and the emergence of Aminicenantes and Planctomycetes, which were less than 1% of the population in the control group. Bacterial communities in the water of the coal reservoir in the study area were more susceptible to fracturing than archaeological communities.

### 3.2. Microbial Evidence for Coal Biosolubilization and Methanogenesis

Previous research and sequencing analyses have shown that most microorganisms can participate in the degradation process of organic matter in coal reservoirs [24,25,26]. Consistent with previous studies, the species of bacteria in the study area were rich, including various heterotrophic microbial types common in nature. In contrast, the species of archaea are relatively small, mainly methanogens. There is a series of microbial communities of coal degradation in the reservoir of the study area [6,12].

Like other coal reservoir microbial studies, *Proteobacteria* was the most abundant microbial type in the reservoir water, including *Alphaproteobacteria*, *Betaproteobacteria*, *Gammaproteobacteria*, and *Deltaproteobacteria*, which contains most organic matter-degrading bacteria [7,17,19]. *Firmicutes* can include types of bacteria that can demethyl aromatic hydrocarbons. The bacteria in *Actinobacteria* are filamentous bacteria that can hydrolyze lipids in coal seams [6,12]. *Bacteroidetes* contain microbial types that can degrade polysaccharides, proteins, and cellulose. Bacteria in *Spirochaetes* can degrade polymers and carbohydrates in an anaerobic environment [8,24].

Acetogens are commonly found in reservoir water. Propionic acid, butyric acid, and lactic acid produce acetic acid and H_2_. For example, Clostridium and Enterobacter may also have the ability to produce acetic acid and hydrogen. They may be more susceptible to environmental factors such as temperature and pH than methanogens [6,27]. Bacillus was detected in all water samples that can produce surfactants to increase the thermal stability of cellulase and promote the degradation of lignocellulose. Its decomposition products can be used as a substrate by *Clostridium* [14]. *Desulfovibrio* is a sulfate-reducing bacteria that cannot completely decompose organic matter into acetic acid. *Desulfovibrio* participates in the carbon and sulfur cycles in the underground environment. Metabolic H_2_S can combine with ferrous ions to form insoluble iron sulfide [17]. *Desulfovibrio* and Bacillus can use iron as an electron acceptor. Bacillus can also use manganese as an electron acceptor, but its efficiency in using metal elements to oxidize organic compounds remains to be studied. *Exiguobacterium Pseudomonas* can produce extracellular compounds to break down various organic substances. *Acinetobacter* can degrade a variety of aromatic compounds. *Klebsiella* sp. can degrade polycyclic aromatic hydrocarbons, carbazole, and other substances. *Pseudomonas* and *Citrobacter* can cooperate with hydrogen-producing bacteria to degrade polycyclic aromatic hydrocarbons and toluene into acetate, H_2_, and CO_2_ [15]. *Enterobacter*, *Clostridium*, and *Klebsiella* have hydrogenase and may also have hydrogen production capacity. Aerobic Hydrogenophaga should not be detected in anaerobic coal seams, but some studies have shown either that it has an anaerobic metabolic capacity or that coal seams are not entirely anaerobic [8]. *Geobacter* and methanogens can efficiently degrade monocyclic aromatic hydrocarbons and long-chain alkanes and produce methane. *Rhizobium* is often active in anaerobic environments and may participate in the nitrogen cycle. The presence of these bacteria provides a substrate for methanogens and is conducive to methanogenesis; thus, the metabolic efficiency of bacteria is critical in the process of degradation of organic matter in coal to methanogenesis [13]. *Nitrosopumilus* has also been found in coal basin reservoirs in China, Japan, and Germany [10,20,28]. *Nitrosopumilus* is relatively common in the marine environment, and its primary metabolism is completed through aerobic ammonium oxidation [10,28]. The genera *Azoarcus* and *Thauera* have polycyclic aromatic hydrocarbon (PAH) dioxygenases, which can degrade aromatic compounds in anaerobic environments, and their role in the biological solubilization process of coal cannot be underestimated [29,30]. 

### 3.3. Environmental Factors Affecting Microbial Composition

**Figure 6:**
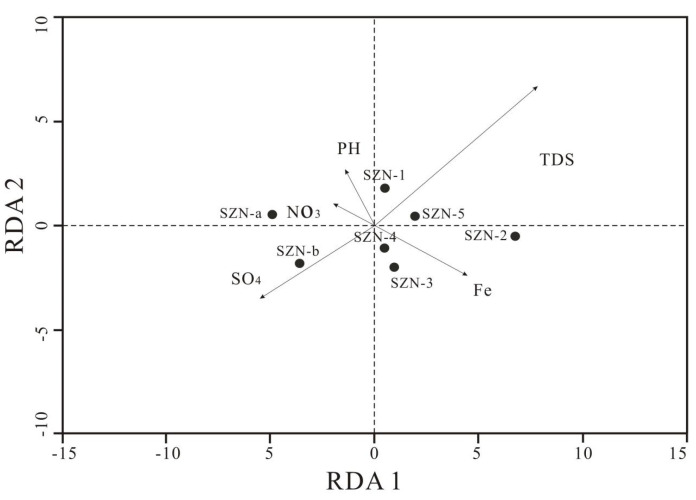
RDA based on microbe level with several important environmental impact factors in the coal reservoir.

RDA was used to study the relationship between environmental factors and microbial structures. Data revealed that the microbial communities in this study were affected by pH, TDS, Fe^3+^, SO_4_^2−^, and NO_3_^−^ to some extent. As shown in Figure 6, the longer the environmental impact factor was, the higher its contribution was. When the environmental impact factor and the sample formed a sharp angle, there was a positive correlation between them. When the environmental impact factor and the sample formed an obtuse angle, there was a negative correlation between them. The correlation between reservoir microorganisms in the study area and TDS and SO_4_^2−^ is significant. Control group samples (SZN-1, SZN-2, SZN-3, SZN-4, and SZN-5) positively correlated with TDS, whereas fracturing group samples (SZN-a, SZN-b) weakened the influence of TDS. As an essential element and electron acceptor involved in microbial metabolism, iron is essential for controlling and fracturing microorganisms. Sulfate significantly impacts microorganisms more than nitrate, indicating that sulfate reduction as an electron acceptor is widespread in reservoir environments [9,11]. Methane-oxidizing organisms may harm the metabolism of methanogens. Because the geographical location of the sampling point and the depth of the coal seam were similar, the effect of pH on microorganisms in the reservoir can be ignored. The temperature of the water samples measured at the sampling points did not differ much and is not discussed here.

Underground environmental factors are essential factors affecting microbial metabolism and enzyme activity. First of all, the underground environment is the ideal environment for microbial communities to survive and a place for energy and material exchange. It is directly related to the abundance, diversity, and metabolic function of microbial communities and also affects the ability of microorganisms to metabolize organic substances in the reservoir environment. Groundwater runoff conditions are related to the redox conditions of the reservoir environment and have an obvious impact on anaerobic organisms such as methanogens [5,6]. In coal reservoirs, runoff from surface water or atmospheric precipitation may improve microorganism nutrition and benefit the diversity of microbial communities. However, the introduction of dissolved oxygen may also change reservoir conditions, and oxidation conditions are conducive to the reproduction and metabolism of aerobic microorganisms. The ion composition of groundwater is not only related to the metabolism of microorganisms, but its concentration is also closely related to the redox environment of coal seams. TDS, SO_4_^2−^, NO_3_^−^, and HCO_3_^−^ can be used to assess groundwater sealing. HCO_3_^−^ is the product of SO_4_^2−^ desulfurization. Low SO_4_^2−^, high HCO_3_^−^, and TDS can be used as evidence of good sealing or relative reduction of the underground environment [31]. Proper temperature, pH, and trace elements are critical to improving metabolic efficiency. Metal elements can affect the growth of microbial communities by regulating the metabolic function of cells or the production of enzymes within a specific range [8].

Fe significantly influences the metabolism of hydrogen-producing bacteria, which may participate in hydrogenase synthesis. The abundance and diversity of hydrogen-producing bacteria will increase with the increase of Fe concentration, but excessive Fe will also poison microorganisms and inhibit the activity of metalloenzymes [9]. As shown in Figure 4, fermentative and hydrogen-producing bacteria such as *Citrobacter*, *Clostridium*, *Enterobacter*, and *Klebsiella* existed in the reservoir of the study area, and their abundance and metabolic function are related to iron. Microorganisms can improve material exchange with the environment to maintain metabolism. Microorganisms can share resources through cooperation, symbiosis, or competition to maintain their survival, reproduction, and metabolic functions. Acetic acid-producing bacteria can use hydrogen to produce acetic acid. They compete with hydrogenotrophic methanogens for hydrogen and provide metabolic substrates for acetoclastic methanogens. Hydrogen-producing bacteria such as *Clostridium* and *Tissierella* provide metabolic substrates. Some hydrolysis and fermentation bacteria can regulate the production of hydrogen and acetic acid through enzymes, cofactors, and metabolic signals [6]. Therefore, the biomethane production pathway in the study area is affected by the production and consumption of available methanogen substrates such as acetic acid and hydrogen.

The appearance of methylotrophic *Methanolobus* in fracturing wells showed that fracturing has changed the metabolic path and intensity of microorganisms, and the methyl substances produced provide the substrate for *Methanolobus*. The study area also had *Staphylococcus*, which is common in the marine environment and has anti-corrosive effects on heavy metals such as manganese [14,15]. *Staphylococcus* in coal reservoirs may also participate in the catabolism of coal and other organic substances. Different types of methanogens exist in the study area, particularly methylotrophic ones in fracturing wells [13]. *Desulfovibrio* and other sulfate-reducing bacteria have more metabolic advantages than hydrogenotrophic methanogens in the case of more sulfates but do not compete with methylotrophic methanogens [17]. After hydraulic fracturing, the metabolism of hydrogen-consuming bacteria may be enhanced [12]. They reduce the hydrogen partial pressure in the system through interspecific hydrogen transfer and promote hydrogen metabolism and hydrogenotrophic methanogenesis [14]. In the reservoir without biomethane, fermenting bacteria, methanogens, and other microorganisms could also be detected. Metabolic intensity is the main factor affecting biomethane production. Hydraulic fracturing stimulates the metabolic intensity of some microorganisms to some extent [32,33].

### 3.4. Microbial Communities and Metabolism Shift for Methanogenesis after Hydraulic Fracturing

Microbial communities with low diversity also have the potential to degrade coal and produce methane, but their efficiency and sustainability need to be discussed. Hydraulic fracturing significantly increases the variety of microorganisms in reservoir water [12,34]. The most obvious difference between water samples collected from fracturing wells (SZN-a, SZN-b) was that the abundance of the main *Actinobacteria* increased at the phylum level as well as the concentration of *Amicenantes* and *Planctomycetes* (1%). *Amicenantes* and *Planctomymeters* comprised less than 1% of the control group’s population. After hydraulic fracturing, the impact on the reservoir’s microbial communities took place within months. Compared to the microbial composition in the control group water sample, the differences in microbial communities in the fracturing well reservoir water may have been caused by the carbon source/nutrients in the fracturing fluid. Changes in the microbial composition of the reservoir caused by hydraulic fracturing fluid, especially the enrichment of *Planctomycetes* and *Amicenantes*, may also be related to the evolution of the physical properties of the coal seams caused by hydraulic fracturing. *Planctomycetes* exist in fresh water, seawater, soil, and other environments rich in organic matter, and they can degrade xylose polymer [6,12]. *Aminocenantes* can degrade various complex organic compounds [13,14,15]. *Aminocenates* contain enzymes with high carbohydrate activity, which are suitable for using a variety of complex organic substances as carbon sources. *Aminicetes* do not use nitrate/nitrite but sulfate as an electron acceptor for aerobic/anaerobic action. *Aminocene*’s oxidizable organic compounds are formate, hydrogen, and carbon dioxide, which provide available substrates for hydrogenotrophic methanogens [8,15]. Other studies have shown that *Aminocenantes* cooperates with hydrogenotrophic methanogens to metabolize acetic acid to produce methane. Therefore, hydraulic fracturing improves the abundance of *Aminicenantes* and is conducive to hydrogenotrophic methanogenesis [8,13,14,15]. Methylotrophic *Methylophaga* can only use methanol and other single carbon supplements. The increase in *Methylphaga* abundance in fracturing wells also indicates that methyl compounds are beginning to appear in the reservoir, which is consistent with the occurrence of methyl methanogens in fracturing wells.

The presence of specific aerobic microorganisms in the underground environment proves that oxygen exists in the reservoir. Aerobic microorganisms are more abundant in the fracturing well near the fault zone. However, with regard to the research design and sampling process, aerobic microorganisms and oxygen may be mixed in the drainage pipes of coalbed methane wells. However, the water pipe environment is unlikely to provide nutrients for microorganisms. These effects can be ignored due to the high flow rate of water in the pipeline and the short sampling time. In other coal reservoirs, there are cases of *ANME* (*Anaerobic Methanototropicarchaea*) discovery [6,31]. *ANME* was not found in the study area, but sulfate-reducing, nitrifying, and other microorganisms exist. The anaerobic oxidation of organic matter and methane may also be coupled with sulfate, nitrate, iron, or manganese. The fact that sulfate decreased with depth in the study area also proves that microbial anaerobic oxidation exists in the study area [35,36]. Methanogens were widely distributed in the study area.

The sequencing results and analysis show that the reservoir’s microbial communities will respond to hydraulic fracturing interference and other factors. First, the abundance of microbial communities associated with organic degradation increased. Increasing the abundance of microbial communities is conducive to continuously improving the metabolic intensity of microbial communities, such as encouraging coal degradation and methanogenesis in the anoxic environment [5,6]. However, fracturing introduces electronic receptors such as oxygen, sulfate, and nitrate. The enhanced metabolism of aerobic or anaerobic methanogens may also consume residual biomethane and thermal methane in coal seams. Hydraulic fracturing may strengthen the carbon circulation process of the reservoir, thereby enhancing the methane circulation that may occur in the block [5,32]. The persistence of hydraulic fracturing on the biogeochemical process of the underground environment can be maintained for several months or even longer. With the passage of time after fracturing, the redox conditions of the reservoir return to their natural state, and the composition and metabolism of microbial communities have substantial changes compared to those before fracturing. Studying the regional biogeochemical cycle at different periods before and after reservoir reconstruction is conducive to studying microbial abundance, diversity, and metabolic function, which is related to the carbon cycle, utilization, and transformation of coal reservoirs in the region [32]. In addition, it is helpful to clarify the path of microbial communities to obtain energy from coal reservoirs via the nitrogen cycle, sulfur cycle, and other aspects to describe the whole cyclical mechanisms of microbial communities and environmental systems related to the underground environment and the cycle process of nitrogen, sulfur, and other life elements. Using culture-independent macrogenomics and other methods can provide various means for studying specific microbial metabolism processes in coal reservoirs. Linking microbial metabolic potential with regional systems requires more in-depth research on microbial activity, function, and nutrient cycles to overcome the biogeological bottleneck of CBM bioengineering.

## 4. Conclusions

This study reveals that the microbial composition of the hydraulic fracturing coal reservoir changed as a result of that fracturing. The diversity of microbial communities related to organic degradation was improved. The proportion of *Actinobacteria* in Shizhuangnan Block reservoir water increased significantly, and the abundance of *Aminicenantes* and *Planctomycetes*, which did not exist in non-fracturing coalbed methane wells or existed at very low abundance, increased. Microbial communities were correlated with environmental factors such as TDS and Fe^3+^. Ecological conditions and geochemical characteristics can lead to corresponding changes in microbial community metabolism. These environmental factors also determined the fermentation and metabolic pathways of methanogens in coal seams. For example, fermentation and hydrogen-producing bacteria such as *Citrobacter*, *Clostridium*, *Enterobacter*, and *Klebsiella* existed in the reservoir in the study area.

There were different types of methanogens in the study area, particularly methyl methanogens in fracturing wells. The fractured underground environment provided substrates for methylotrophic methanogens. After hydraulic fracturing, the metabolism of hydrogen-consuming bacteria may be enhanced, which promotes the metabolism of hydrogenotrophic methanogens. Hydraulic fracturing stimulates the metabolic intensity of some microorganisms to some extent. After hydraulic fracturing, the impact on the reservoir’s microbial communities remained for months. Compared to the microbial composition in the control group water sample, the differences between microbial communities in fracturing well reservoir water may have been caused by the carbon source/nutrient in fracturing fluid. Microbial communities are generally associated with coal degradation and methane generation in coal reservoirs. The production and consumption of biological metabolic substrates are related to environmental regulation. The symbiotic relationships between microbial communities determine the material cycle of the underground environment. Hydraulic fracturing may enhance the carbon circulation process of the reservoir, thereby enhancing the methane and carbon dioxide circulation that may occur in the block. The relationship of the duration of hydraulic fracturing to the biogeochemical process of the underground environment and the importance of hydraulic fracturing to the biogeochemical cycle need to be further studied.

## Figures and Tables

**Table 1:** Aqueous geochemistry of CBM co-produced water samples in the Shizhuangnan Block.

No.	pH	Cl^−^ (mg/L)	HCO_3_^−^ (mg/L)	NO_3_^−^ (mg/L)	SO_4_^2−^ (mg/L)	Na^+^ (mg/L)	Ca^2+^ (mg/L)	Mg^2+^ (mg/L)	Fe^3+^ (mg/L)	TDS (mg/L)
SZN-1	6.8	219.36	537.84	11.47	3.89	491.39	3.57	3.84	4.84	1276.2
SZN-2	7.5	223.69	328.52	5.32	5.27	684.02	5.86	4.86	2.09	1259.63
SZN-3	7.1	129.37	631.83	6.16	6.85	563.84	2.26	2.27	1.07	1343.65
SZN-4	6.9	247.69	336.09	8.56	7.09	772.06	4.72	1.98	6.75	1384.94
SZN-5	7.2	165.48	296.95	7.63	8.37	684.85	3.83	5.37	3.82	1176.3
SZN-a	7.6	297.96	528.93	6.94	2.47	685.37	6.48	2.86	2.83	1533.84
SZN-b	6.7	262.98	619.05	5.83	4.85	863.08	5.93	3.54	1.63	1766.89
